# Supplementary material for: Quantitative Proteomic Characterization of Foreign Body Response towards Silicone Breast Implants Identifies Chronological Disease-Relevant Biomarker Dynamics
Source: Biomolecules. 2023 Feb 6;13(2):305. doi: 10.3390/biom13020305 (PMC9953687; doi:10.3390/biom13020305)
Supplement: Supplementary file 1 [file biomolecules-13-00305-s001.zip › Supplementary figures.pdf]

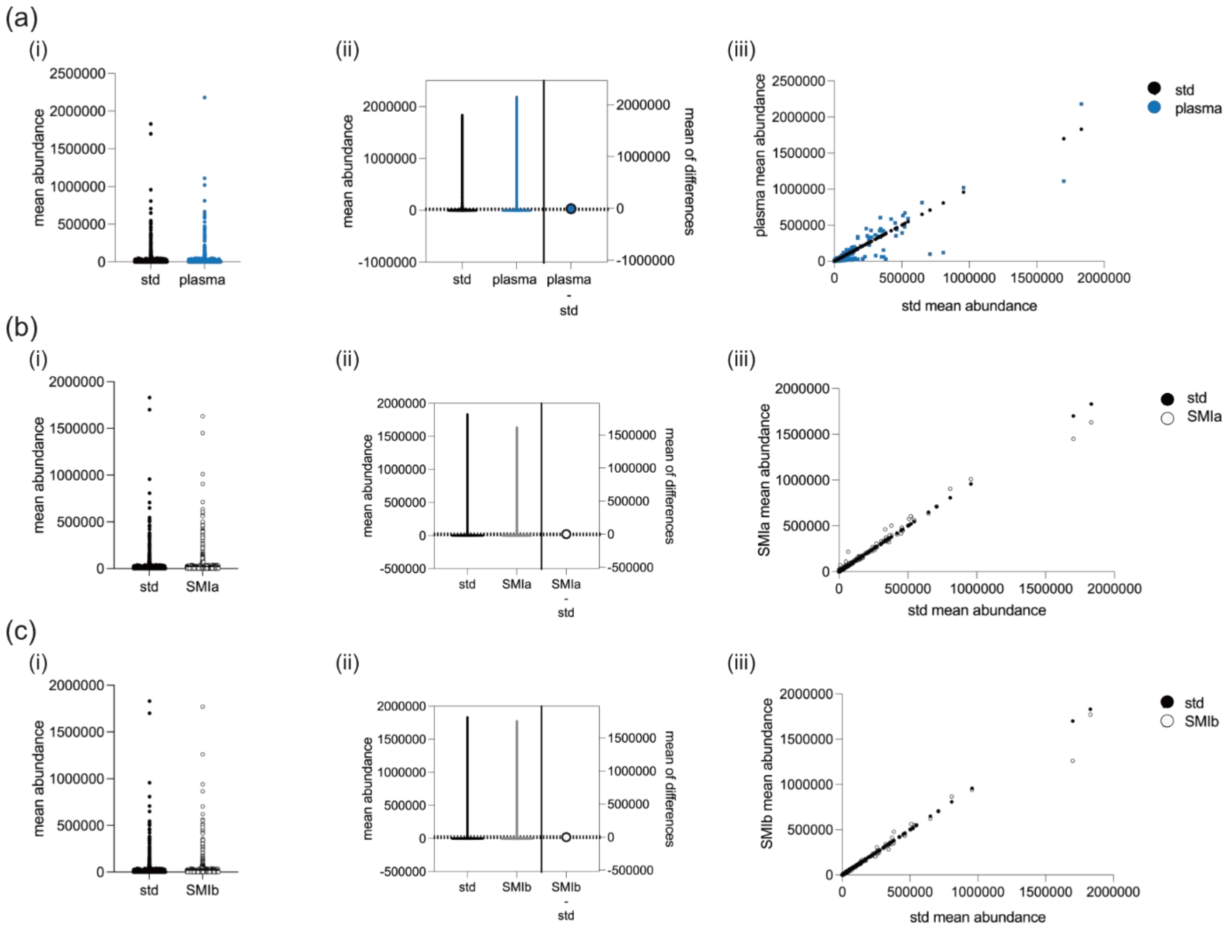

Figure S1. LC-MS/MS reproducibility and distribution of wound proteins identified in **(a)** plasma and wound bed fluid formed around **(b)** SMIa and **(c)** SMIb.

(i) Scatter plot reveals an estimated significant sample difference compared to standard with comparative general protein abundance (ii) and (iii) high correlation coefficient between proteins detected.

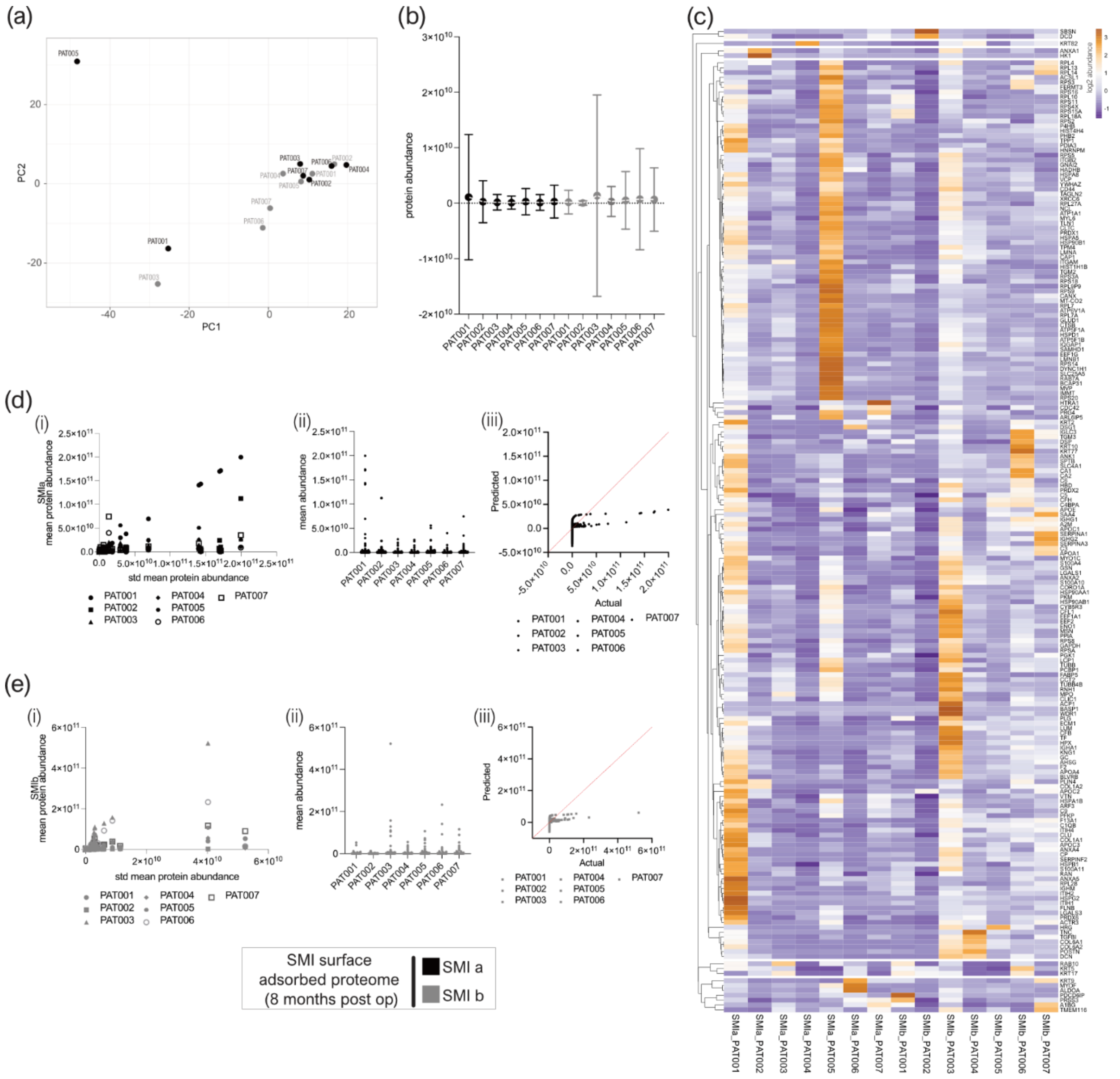

Figure S2. LC-MS/MS reproducibility and distribution of SMI adhesive proteins identified. **(a)** PCA obtained with log2 transformed protein abundance of SMI surface-associated proteins found in all samples by adhesive proteome analysis on 2 different silicone tissue expanders implanted in 7 patients, app. 6- 8 months post-surgery. **(b)** Correlation plot reveals an estimated significant inter- and intra-individual sample variation of stripped SMI adhesive proteome in general protein abundance (one-way Anova: F (13, 14647) = 3,565; R2=0.003154). **(c)** Heatmap analysis SMI surface associated/adhesive proteome. Rows: Clustered by Manhattan distance, average method, and tightest cluster first tree ordering. Columns: no clustering applied. **(d-e)** An interindividual (i) significant patient sample correlation of protein abundance, (ii) significant difference of data sets in both groups (iii) and lognormal distribution for both datasets between proteins detected. The level for statistical significance was set, for all statistical tests. (inter- and intra-individual comparison; n=7)

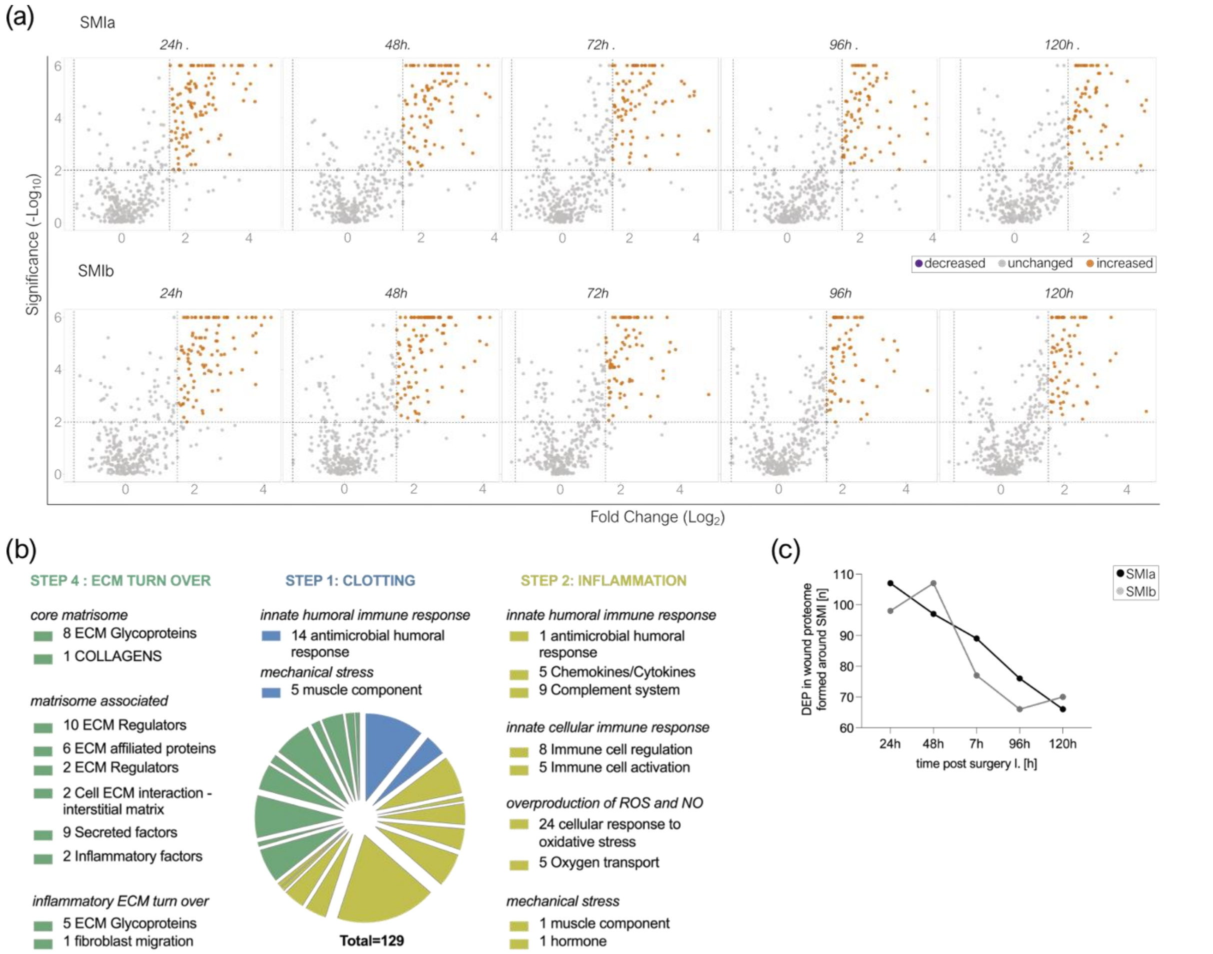

Figure S3. Intraindividual comparative analysis of plasma derived DEPs in the acute wound 1-5 days post SMI implantation.**(a)** Volcano plot of differentially expressed proteins in wound bed fluid formed around SMIa and SMIb (effect threshold [FC<-1.5, FC>1.5] significance threshold > 2), 1-5 days after device implantation compared to untriggered plasma proteome pre-op. **(b)** Functional categorization in context of tissue repair of DEPs in common plasma derived wound proteome formed around 2 different silicone tissue expanders implanted, 1 to 5 days post-surgery. **(c)** Correlation plot of DEP expression 1 to 5 days post-surgery. The level for statistical significance was set, for all statistical tests. (inter- and intra-individual comparison; n=7)

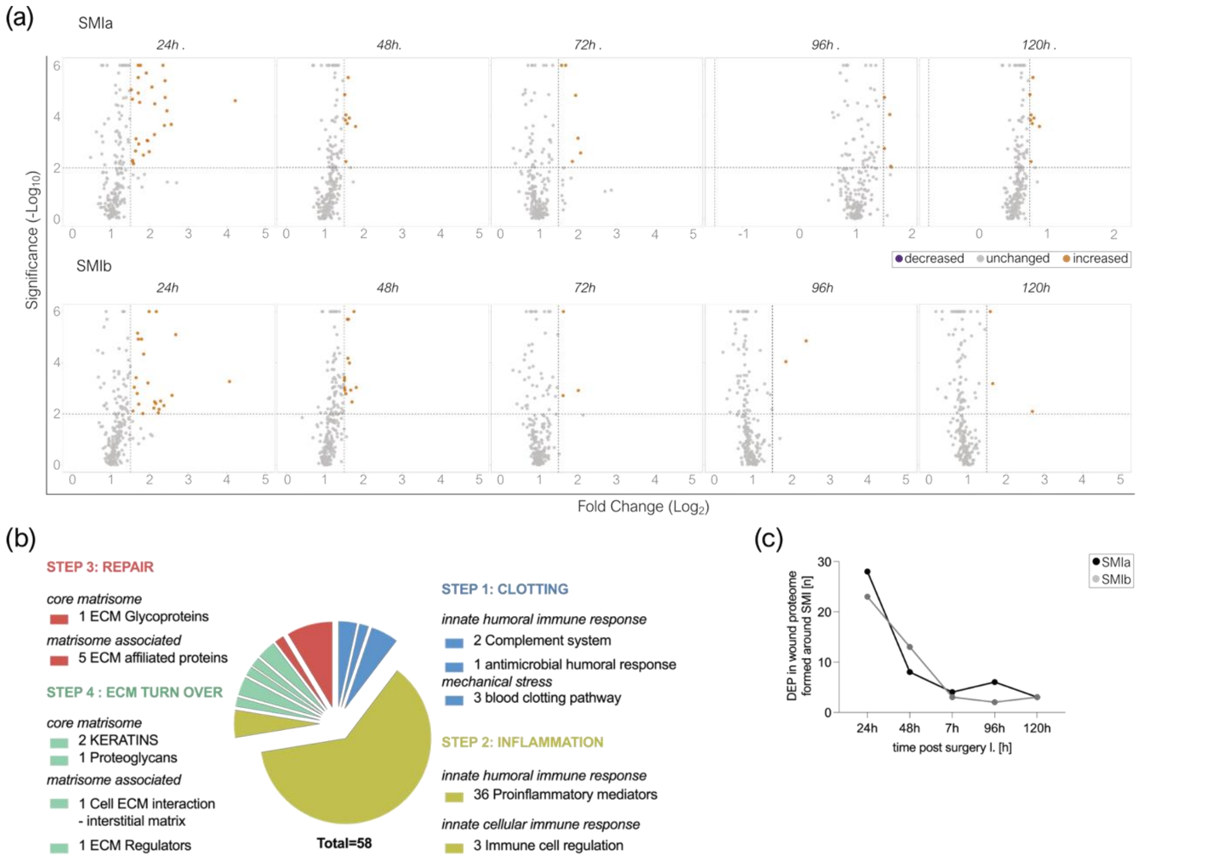

Figure S4: Intraindividual comparative analysis of local DEPs in the acute wound 1-5 days post SMI implantation **(a)** Volcano plot of differentially expressed local wound proteins in wound bed fluid formed around SMIa(i) and SMIb (ii) 1-5 days after device implantation compared standard control (effect threshold [FC<-1.5, FC>1.5] significance threshold > 2). **(b)** functional categorization in context of tissue repair of DEPs in common local wound proteome formed around 2 different silicone tissue expanders implanted, 1 to 5 days post-surgery. **(c)** Correlation plot of DEP expression 1 to 5 days post-surgery. The level for statistical significance was set, for all statistical tests. (inter- and intra-individual comparison; n=7)

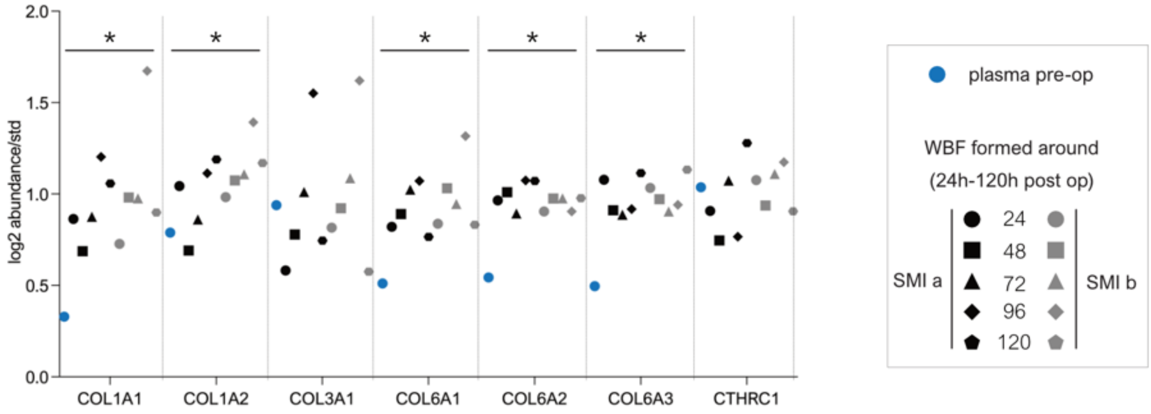

Figure S5. Comparative analysis of collagen abundance in plasma pre-op and wound bed fluid 1-5 days post implantation.
